# Coronavirus disease 2019 (COVID-19) and QTc prolongation

**DOI:** 10.1186/s12872-021-01963-1

**Published:** 2021-03-30

**Authors:** Khalid Changal, David Paternite, Sean Mack, Spiro Veria, Rehana Bashir, Mitra Patel, Ronak Soni, Muhammad Ali, Tanveer Mir, Mujeeb Sheikh, P. Kasi Ramanathan

**Affiliations:** 1 Cardiovascular Medicine, University of Toledo, Toledo, OH USA; 2 University of Toledo College of Medicine and Life Sciences, Toledo, USA; 3 Owen’s Community College, Toledo, OH USA; 4 Internal Medicine, University of Toledo, Toledo, OH USA; 5 Internal Medicine, Detroit Medical Center, Wayne State University, Detroit, MI USA; 6 Department of Cardiovascular Medicine and Interventional Cardiology, Promedica Toledo Hospital, 2109 Hughes Dr, Jobst Tower 3rd, Floor, Toledo, OH 43606 USA

**Keywords:** QT prolongation, COVID-19, Cardiovascular disease

## Abstract

**Introduction:**

The cause-and-effect relationship of QTc prolongation in Coronavirus disease 2019 (COVID-19) patients has not been studied well.

**Objective:**

We attempt to better understand the relationship of QTc prolongation in COVID-19 patients in this study.

**Methods:**

This is a retrospective, hospital-based, observational study. All patients with normal baseline QTc interval who were hospitalized with the diagnosis of COVID-19 infection at two hospitals in Ohio, USA were included in this study.

**Results:**

Sixty-nine patients had QTc prolongation, and 210 patients continued to have normal QTc during hospitalization. The baseline QTc intervals were comparable in the two groups. Patients with QTc prolongation were older (mean age 67 vs. 60, P 0.003), more likely to have underlying cardiovascular disease (48% versus 26%, P 0.001), ischemic heart disease (29% versus 17%, P 0.026), congestive heart failure with preserved ejection fraction (16% versus 8%, P 0.042), chronic kidney disease (23% versus 10%, P 0.005), and end-stage renal disease (12% versus 1%, P < 0.001). Patients with QTc prolongation were more likely to have received hydroxychloroquine (75% versus 59%, P 0.018), azithromycin (18% vs. 14%, P 0.034), a combination of hydroxychloroquine and azithromycin (29% vs 7%, P < 0.001), more than 1 QT prolonging agents (59% vs. 32%, P < 0.001). Patients who were on angiotensin-converting enzyme inhibitors (ACEi) were less likely to develop QTc prolongation (11% versus 26%, P 0.014). QTc prolongation was not associated with increased ventricular arrhythmias or mortality.

**Conclusion:**

Older age, ESRD, underlying cardiovascular disease, potential virus mediated cardiac injury, and drugs like hydroxychloroquine/azithromycin, contribute to QTc prolongation in COVID-19 patients. The role of ACEi in preventing QTc prolongation in COVID-19 patients needs to be studied further.

## Highlights

- QTc prolongation is common in COVID-19 patients in a hospital setting.
- Older age, ESRD, underlying cardiovascular disease, potential virus mediated cardiac injury, and drugs like hydroxychloroquine/azithromycin, contribute to QTc prolongation in COVID-19 patients.
- QTc prolongation is related to adverse patient outcomes in COVID-19.
- Angiotensin-converting enzyme (ACE) inhibitors need to be studied further for their role in preventing QTc prolongation in COVID-19 patients.

## Introduction

In December 2019, the novel coronavirus–infected pneumonia (NCIP), started in Wuhan, China and spread worldwide to cause a pandemic [1–5]. Coronavirus disease 2019 (COVID-19) continues to affect the United States of America (USA) severely. As of 27 December 2020, the number of cases in the USA has reached 18,730,806, the most of any country in the world. The number of deaths is 329,592 and continues to increase [6]. While the predominant reason for mortality and morbidity is respiratory involvement and failure [7]. The cardiovascular effects of COVID-19 include cardiac injury, myocarditis, cardiomyopathy, heart failure, arrhythmias, and thromboembolic phenomena [8–10]. QTc prolongation has been noticed in hospitalized COVID-19 patients. The cause-and-effect relationship of QTc prolongation in COVID-19 patients has not been studied well. We attempt to study this relationship in this retrospective study.

## Methods

This is a retrospective, hospital-based, observational study. All patients who were hospitalized with the diagnosis of COVID-19 infection from 1 January 2020 through 1 May 2020 at two tertiary care hospitals in Toledo, Ohio were included in this study. Real-Time RT-PCR (cobas® SARS-CoV-2 Test) was utilized for diagnosis in nasopharyngeal and oropharyngeal swab samples from patients. Patients less than 18 years old and those with history of QTc prolongation were excluded. History of QTc prolongation was defined as prolongation of QTc on an EKG done (when available) at least 4 weeks prior. If prior EKG was not available (18% patients) the QTc was presumed to have been normal. All patients had an admission EKG. The normal QTc in men is less than 450 ms and less than 470 ms in women [11]. For the purpose of the study and to include true positive cases, QTc was considered prolonged if more than 460 ms in men and more than 480 ms in women on any EKG done during hospital stay. This cut off was used to allow for variations in measurement.

We collected data on demographics, comorbidities, electrocardiography (EKG), corrected QT interval, length of hospital stay, cost of hospitalization, in-patient mortality, and other clinical outcomes. The data was collected by reviewing the electronic medical records of the patients. The hospital system maintains elaborate medical records of all patients treated. We used Bazett’s formula for measuring QTc. The QT intervals were measured manually using the tangent method and dividing by the square root of the RR interval preceding the QT interval. For patients with intraventricular conduction delays (bundle branch block, paced rhythms), a modified QTc was calculated using the formula: (QT—(QRS—120msec)) /√RR [12]. All the EKGs done during the hospital stay were studied.

The data were analyzed using Statistical Package for the Social Sciences (SPSS), Version 20.0, from SPSS incorporation Chicago, IL. We used mean, standard deviation/standard error of mean, and percentage when appropriate for the patient’s characteristic description. Group differences were compared using the Pearson *χ*2 or Fisher's exact test for categorical variables, or the Student *t* test or the Mann–Whitney *U* test for continuous variables. *P*-values of 0.05 or less were considered statistically significant. We conducted univariate and multivariate regression analysis for factors contributing to QTc prolongation, and for the effect of QTc prolongation on different outcomes. In multivariate analysis, only the variables with statistical significance on univariate analysis were studied. Multivariate analysis was done separately for baseline comorbidities (7 variables) and for hospital course/clinical outcomes (7 variables).

The study was approved by the Institutional Review Board of Promedica Health System in Toledo, Ohio, USA.

## Results

A total of 279 patients were included in this study. Sixty-nine patients met the inclusion criteria of QTc prolongation, and 210 patients were included in the normal QTc group. The average baseline QTc on admission in all patients was 430 ± 18 ms. The baseline QTc in the two groups was comparable (431 ± 20 vs. 430 ± 19 ms, P 0.72). The mean longest QTc in the QT prolongation group was 492 ± 27.2 ms (range 462–600), and in the normal QT group was 431 ± 25.7 ms (294 – 471). Baseline characteristics are described in Table 1. Patients with QTc prolongation were older (mean age 67 versus 60 years, P 0.003), more likely to have underlying cardiovascular disease (48% versus 26%, P 0.001), ischemic heart disease (29% versus 17%, P 0.026), congestive heart failure with preserved ejection fraction (16% versus 8%, P 0.042), chronic kidney disease (23% versus 10%, P 0.005), and end-stage renal disease (ESRD) (12% versus 1%, P < 0.001). Patients who were on angiotensin-converting enzyme inhibitors (ACEi) were less likely to develop QTc prolongation (11% versus 26%, P 0.014). No significant statistical difference was noticed for gender, race, hypertension, congestive heart failure with reduced ejection fraction, history of atrial fibrillation, stroke, or chronic liver disease.

**Table 1:** Patient characteristics and comorbidities; comparison of patients with and without QTc prolongation on EKG

Baseline characteristics	All patients (N = 279)	QTc prolongation (N = 69)	No QTc prolongation (N = 210)	P value
Baseline QTc (ms)	430 ± 18	431 ± 20	430 ± 19	0.72
Age	62 ± 17	67 ± 17	60 ± 17	0.003
*Sex*				
Male, n (%)	145 (52)	39 (57)	106 (51)	0.383
Female, n (%)	134 (48)	30 (44)	104 (50)	
*Race*				
Caucasian, n (%)	181 (65)	40 (58)	141 (68)	0.097
African-American, n (%)	82 (30)	27 (39)	55 (26)	
Latino, n (%)	13 (5)	1 (1)	12 (6)	
Other, n (%)	2 (1)	1 (1)	1 (1)	
Hypertension, n (%)	197 (70)	53 (77)	144 (67)	0.192
Diabetes mellitus, n (%)	101 (36)	31 (45)	70 (33)	0.082
Cardiovascular disease, n (%)	87 (31)	33 (48)	54 (26)	0.001
Ischemic heart disease, n (%)	55 (20)	20 (29)	35 (17)	0.026
CHFrEF, n (%)	13 (5)	5 (7)	8 (4)	0.240
CHFpEF, n (%)	27 (10)	11 (16)	16 (8)	0.042
Atrial fibrillation, n (%)	28 (10)	11 (16)	17 (8)	0.060
Active cancer, n (%)	12 (4)	3 (4)	9 (4)	0.982
Stroke, n (%)	33 (12)	8 (12)	25 (12)	0.945
Chronic kidney disease, n (%)	37 (13)	16 (23)	21 (10)	0.005
ESRD on HD, n (%)	11 (4)	8 (12)	3 (1)	< 0.001
Chronic liver disease, n (%)	13 (5)	6 (9)	7 (3)	0.062
Immunosuppressive state, n (%)	19 (7)	7 (10)	12 (6)	0.193
Home med: ACEi, n (%)	62 (22)	8 (11)	54 (26)	0.014
Home med: ARBs/ARNI, n (%)	32 (12)	8 (12)	24 (11)	0.970

CHFpEF = congestive heart failure with preserved ejection fraction, CHFrEF = congestive heart failure with reduced ejection fraction, ESRD on HD = End stage renal disease on hemodialysis, Immunosuppressive state = anyone on chronic immunomodulatory drugs or with immunodeficiencies such as HIV, ARNI = Angiotensin Receptor-Neprilysin Inhibitor, EKG = Electrocardiogram

Table 2 describes the effect of QTc prolongation on hospital course and clinical outcomes. Patients with QTc prolongation were more likely to have another EKG abnormality also (84% versus 21%, P < 0.001), first degree heart block (6% versus 1%, P 0.016), new left or right bundle branch block (20% vs. 5% P < 0.001), elevated troponin I (32% versus 18%, P 0.021), type 2 myocardial infarction (16% versus 7%, P 0.029), and elevated creatinine (average of 2.5 mg/dL versus 1.6, P 0.004). Patients with QTc prolongation were more likely to have received hydroxychloroquine (75% versus 59%, P 0.018), azithromycin (18% vs. 14%, P 0.034), a combination of hydroxychloroquine and azithromycin (29% vs 7%, P < 0.001), more than 1 QT prolonging agent (59% vs. 32%, P < 0.001). Patients with QTc prolongation were less likely to be discharged home and more likely to be discharged to a skilled nursing facility. Patients who continued to take ACEi while admitted were less likely to have QTc prolongation (7% vs 18%, P 0.037). There was no association between QTc prolongation and cardiac arrest, ventricular tachycardia, abnormal BNP, elevated d-dimer, hypokalemia, hypomagnesemia, shock, acute respiratory distress syndrome, length of stay, cost of hospitalization, or death.

**Table 2:** Patient outcomes and laboratory studies during hospitalization, and comparison of patients with and without QTc prolongation on EKG

Clinical course/outcome	All patients (N = 279)	QTc prolongation (N = 69)	No QTc prolongation (N = 210)	P value
Longest QTc measurement (msec)	446 ± 37	492 ± 27.2 (462–600)	431 ± 25.7 (294–471)	< 0.001
Cardiac arrest, n (%)	1 (0.4)	0	1 (0.5)	0.566
*EKG & cardiac rhythm abnormalities*				
Atrial fibrillation, n (%)	25 (9)	9 (13)	16 (8)	0.175
Sustained VT, n (%)	4 (1)	2 (3)	2 (1)	0.240
1st degree heart block, n (%)	6 (2)	4 (6)	2 (1)	0.016
2nd (Type 2) or 3rd degree heart block, n (%)	2 (1)	1 (1)	1 (1)	0.406
New left or right bundle branch block, n (%)	24 (9)	14 (20)	10 (5)	< 0.001
Abnormal EKG, n (%)	102 (37)	58 (84)	44 (21)	< 0.001
Any arrhythmia, n (%)	40 (14)	18 (26)	22 (11)	0.001
*High troponin I, n (%)*	58 (22)	21 (32)	37 (18)	0.021
Troponin I peak (ng/mL)	0.34 ± 1.50	0.86 ± 2.60	0.16 ± 0.88	0.001
*Abnormal BNP, n (%)*	45 (30)	14 (33)	31 (28)	0.640
BNP peak (pg/mL)	185 ± 299	277 ± 427	154 ± 236	0.064
*High d-dimer, n (%)*	193 (73)	54 (82)	139 (70)	0.052
D-dimer peak (ng/mL)	3254 ± 8868	3979 ± 9035	3019 ± 8824	0.463
Acute myocardial infarction (not Type 2), n (%)	2 (1)	0 (0)	2 (1)	0.416
Type 2 myocardial infarction, n (%)	26 (9)	11 (16)	15 (7)	0.029
*Acute kidney injury, n (%)*	111 (40)	34 (49)	77 (37)	0.063
Peak creatinine (mg/dL)	1.85 ± 2.15	2.50 ± 2.90	1.60 ± 1.80	0.004
New HD or CVVHD, n (%)	7 (3)	2 (3)	5 (2)	0.811
Hypokalemia, n (%)	44 (16)	15 (22)	29 (14)	0.117
Hypomagnesemia, n (%)	39 (14)	11 (16)	18 (13)	0.558
Invasive ventilation, n (%)	52 (19)	14 (20)	38 (18)	0.685
Shock of any type, n (%)	45 (16)	10 (15)	35 (17)	0.692
ARDS, n (%)	43 (16)	5 (7)	38 (18)	0.032
Ischemic Stroke, n (%)	2 (1)	0 (0)	2 (1)	0.415
DVT and/or PE, n (%)	11 (4)	2 (3)	9 (4)	0.603
Death, n (%)	42 (15)	14 (21)	28 (13)	0.151
*Discharge*				
Home, n (%)	74 (62)	33 (48)	141 (67)	0.023
SNF, n (%)	60 (22)	21 (30)	39 (19)	
LOS (days)	9 ± 9	10 ± 11	9 ± 8	0.572
Cost of hospitalization (US dollars)	92,973 ± 125,980	94,889 ± 126,219	87,142 ± 125,990	0.658
Hydroxychloroquine, n (%)	177 (63)	52 (75)	125 (59)	0.018
Azithromycin, n (%)	27 (10)	13 (18)	14 (7)	0.034
Hydroxychloroquine AND Azithromycin, n (%)	35 (13)	20 (29)	15(7)	< 0.001
> 1 QT prolonging drug, n (%)	108 (39)	41 (59)	67 (32)	< 0.001
In hospital: ACEi/ARNI, n (%)	42 (15)	5 (7)	37 (18)	0.037
In hospital: ARBs, n (%)	32 (12)	9 (13)	23 (11)	0.636

SI units for BNP (pmol/L) = ng/mL * 3.46

SI units for creatinine (μmol/L) = mg/dL * 88.4

SI units for troponin I (μg/L) = ng/ml * 1

ARNI = angiotensin receptor-neprilysin inhibitor, VT = ventricular tachycardia, ARDS = acute respiratory distress syndrome, PE = pulmonary embolism, HD = hemodialysis, CVVD = continuous venovenous hemodialysis, SNF = skilled nursing facility, LOS = length of stay

Univariate and Multivariate regression analysis were performed and are detailed in Table 3. On univariate analysis the odds of having QTc prolongation were higher with age (OR 1.03, 95% CI 1.01–1.04), underlying cardiovascular disease (OR 2.60, 95% CI 1.51–4.66), ischemic heart disease (OR 2.04, 95% CI 1.08–3.85), congestive heart failure with preserved ejection fraction (OR 2.30, 95% CI 1.01–5.23), chronic kidney disease (OR 2.72, 95% CI 1.33–5.57), end stage renal disease (OR 9.05, 95% CI 2.33–35.16). QTc prolongation was associated with higher odds of having a new left or right bundle branch block (OR 5.09, 95% CI 2.14–12.08), any arrhythmia during hospitalization (OR 3.00, 95% CI 1.49–6.02), elevated troponin (OR 2.08, 95% CI 1.11–3.90), and type 2 myocardial infarction (OR 2.46, 95% CI 1.07–5.66). The odds of developing QTc prolongation were higher with the use of hydroxychloroquine (OR 2.10, 95% CI 1.20–3.80), azithromycin (OR 3.25, 95% CI 1.44–7.32), and combined use of hydroxychloroquine and azithromycin (OR 5.31, 95% CI 2.53–11.10).

**Table 3:** Univariate and multivariate analysis for factors associated with QTc prolongation

Clinical factors	Univariate analysis			Multivariate analysis		
	Odds ratio	95% confidence interval	P value	Odds ratio	95% confidence interval	P value
Age	1.03	1.01–1.04	0.003	1.01	0.99–1.04	0.210
Female sex	0.78	0.45–1.36	0.384	–	–	–
Hypertension	1.52	0.81–2.85	0.194	–	–	–
Diabetes mellitus	1.63	0.94–2.84	0.083	–	–	–
*Cardiovascular disease*	2.65	1.51–4.66	0.001	1.58	0.47–5.38	0.464
Ischemic heart disease	2.04	1.08–3.85	0.027	0.81	0.25–2.66	0.730
CHFrEF	1.97	0.62–6.24	0.248	–	–	–
CHFpEF	2.30	1.01–5.23	0.047	1.03	0.31–3.44	0.965
Atrial fibrillation	2.15	0.96–4.86	0.065	–	–	–
Chronic kidney disease	2.72	1.33–5.57	0.006	1.37	0.55–3.42	0.495
ESRD on HD	9.05	2.33–35.16	0.001	7.70	1.63–36.25	0.010
Peak creatinine	1.17	1.04–1.32	0.008	–	–	–
Chronic liver disease	2.81	0.91–8.66	0.073	–	–	–
*Home medication*						
ACEi	0.38	0.17–0.84	0.017	0.24	0.09–0.63	0.004
ARBs/ARNi	1.02	0.43–2.38	0.970	–	–	–
**Hospital course and clinical outcome**						
*EKG findings*						
Atrial fibrillation	1.81	0.76–4.30	0.180	–	–	–
Sustained VT	3.09	0.43–22.36	0.264	–	–	–
New BBB	5.09	2.14–12.09	< 0.001	5.20	1.72–15.67	0.003
Arrhythmia on admission	0.99	0.90–1.08	0.739	–	–	–
Arrhythmia in hospital course	3.00	1.50–6.02	0.002	1.70	0.43–2.67	0.886
High troponin I	2.08	1.11–3.90	0.022	2.11	0.93–4.80	0.982
Abnormal BNP	1.20	0.56–2.57	0.640	–	–	–
High d-dimer	1.98	0.99–3.95	0.055	–	–	–
Type 2 myocardial infarction	2.47	1.07–5.66	0.033	2.71	0.89–8.24	0.079
Death	1.68	0.82–3.41	0.154	–	–	–
Cost of hospitalization	1.00	1.00–1.00	0.658	–	–	–
In hospital: ACEi	0.37	0.12–0.97	0.043	–	–	–
Hydroxychloroquine	2.10	1.20–3.80	0.019	2.49	1.23–5.02	0.028
Azithromycin	3.25	1.44–7.32	0.041	2.87	1.11–9.23	0.045
Hydroxychloroquine AND Azithromycin	5.31	2.53–11.11	0.026	4.14	1.09–15.72	0.037
> 1 QT prolonging drug	3.03	1.70–5.40	< 0.001	–	–	–

CHFpEF = congestive heart failure with preserved ejection fraction, CHFrEF = congestive heart failure with reduced ejection, ARNI = angiotensin receptor-neprilysin inhibitor, ESRD on HD = end stage renal disease on hemodialysis, VT = ventricular tachycardia, BBB = bundle branch block (complete left or right),

On multivariate analysis the association of QTc prolongation with end stage renal disease, new bundle branch block, use of hydroxychloroquine, azithromycin, and combination use of azithromycin and hydroxychloroquine was confirmed. The odds of having QTc prolongation were lower with the use of ACEi in both univariate and multivariate analysis.

## Discussion

Our study found QTc prolongation in one-fourth of the hospitalized COVID-19 patients. The factors strongly associated with QTc prolongation are ESRD, use of hydroxychloroquine, and/or azithromycin, older age, underlying cardiovascular disease, elevated troponin, type 2 MI, other EKG abnormalities, and new bundle branch block. Use of ACEi decreased the odds of developing QTc prolongation. QTc prolongation was not found to increase the risk of developing ventricular tachycardia or death and was not associated with increased cost of hospitalization. However, QTc prolongation was associated with more discharges to skilled nursing facilities than to home.

Our study indicates that QTc prolongation in COVID-19 patients is likely related to an interplay between patient-related factors, administration of therapeutic agents, and the disease itself. In the absence of definitive curative therapies, hydroxychloroquine and azithromycin have been used to treat COVID-19 patients [13–15]. However, the recent RECOVERY trial (Randomized evaluation of COVID-19 therapy) failed to show any benefit of hydroxychloroquine in COVID-19 patients and on the contrary showed some signals towards harm [16]. Some recent observational studies have also pointed to the association of QTc prolongation in COVID-19 with the use of hydroxychloroquine and/or azithromycin [17–20]. Our study provides further evidence to the association of QTc prolongation with the use of hydroxychloroquine and azithromycin in this population. In the absence of clear benefit, these drugs are being avoided now, especially in patients with other risk factors for QTc prolongation. If they are used, QTc must be monitored closely. This is the first study to demonstrate a protective effect of ACEi on prolongation of QTc in COVID-19. While there is evidence to suggest that ACEi may reduce QT dispersion in heart failure and reduce QT interval in hypertension [21, 22], the effect of ACEi on QTc in COVID-19 has not been studied. In animal models, rats with hypertension had reduction in QT interval with ACEi but not with calcium channel blockers [22]. Previous studies showed ACEi increase the expression of ACE2, a cellular receptor for SARS-CoV-2 [23–25]. COVID‐19 causes a severe acute respiratory syndrome by binding target epithelial lung cells through angiotensin‐converting enzyme 2 (ACE2) in humans, provoking a concern that the use of ACEi might lead to increased mortality and severity of COVID-19 [26, 27]. Recent evidence has disproven this concern [25]. Patients with hypertension with COVID‐19 could have worse prognosis. The benefits of ACE inhibitors/ARBs in COVID‐19 could outweigh the risks and should not be withheld [27]. Whether the effect of ACEi on QTc in COVID-19 can be replicated in larger studies, and the mechanism involved needs to be evaluated. The benefits of ACE inhibitors/ARBs in COVID‐19 could outweigh the risks and should not be withheld [27].

The possible patient-related factors associated with QTc prolongation in our study include older age, ESRD, and underlying cardiovascular disease. Older age was found to be associated with QTc prolongation in another study also [20]. ESRD was associated QTc prolongation. This can be explained by poor renal excretion of hydroxychloroquine in these patients [28, 29]. Although, hypocalcemia was not studied by us, this could be seen in ESRD and may contribute to QTc prolongation. The association of troponin elevation and type 2 MI can be due to ischemia contributing to QTc prolongation. We found diabetes mellitus in more patients with QTc prolongation than those with normal QTc but was not statistically significant. Diabetes is a poor prognostic factor for patients with COVID-19 [30, 31]. The (chronic and acute) hyperglycemia could negatively affect clinical outcomes and reduce the response to anti-SARS-COV2 therapies [32, 33]. In patients without viral disease and stable condition, the hyperglycemia is a negative factor to cause prolonged QTc interval with alteration of ventricular refractoriness and ventricular arrhythmias [34]. The overall increased risk of arrhythmias and other EKG abnormalities point to electrical instability of the myocardium in COVID-19 patients. There seems to be a complex interplay of multiple factors contributing to or arising from QTc prolongation.

The study did not find an association between torsades de pointes and QTc prolongation. Considering the sample size, the study is likely underpowered to determine this. Other observational studies also have failed to show this association but are limited by small sample size [15–19].

The pathophysiology of cardiac involvement in COVID-19 infection has been thought to be due to direct invasion of virus into the cardiac tissue, and indirectly from the effects of inflammation. SARS-CoV binds to cells expressing appropriate viral receptors, particularly ACE2, which is abundant in the heart [10].

This study has certain limitations. This is an observational study which is prone to multiple biases. However, in the absence of randomized control trials and in the presence of an ongoing pandemic this study provides useful information. There was no preformed protocol for QTc monitoring in these patients and the decision relied on physician judgement. The patients who received QTc prolonging medications could have had their QTc interval monitored more closely than others. However, on our chart review we found uniform number of EKGs done in most patients. All patients in this study had continuous telemetry monitoring. Long-term follow-up is not available for these patients as this study was done on a hospitalized patient population.

To conclude, QTc prolongation is common in COVID-19 patients. Interplay of multiple factors possibly contribute to this phenomenon. Hydroxychloroquine and azithromycin cause QTc prolongation in this patient population. Older age, ESRD, and underlying cardiovascular disease are risk factors for QTc prolongation. ACEi may prevent QTc prolongation in COVID-19 patients and this needs to be studied further.

## Data Availability

The datasets used and/or analysed during the current study available from the corresponding author on reasonable request.
